# Mitochondrial DNA homeostasis: A novel therapeutic target for neurodegenerative diseases

**DOI:** 10.4103/NRR.NRR-D-25-00495

**Published:** 2025-09-29

**Authors:** Tingting Fu, Xinyi Chen, Shuting Zhang, Ying Fu, Ling Huang, Wandi Xiong

**Affiliations:** Key Laboratory of Tropical Biological Resources of Ministry of Education, School of Pharmaceutical Sciences, Hainan University, Haikou, Hainan Province, China

**Keywords:** aging, Alzheimer’s disease, heteroplamy, mitochondrial DNA mutation, mitochondrial DNA, mitochondrial genome, mitochondrial haplogroup, mitochondrial homeostasis, neurodegenerative diseases, Parkinson’s disease

## Abstract

The mitochondrial genomic homeostasis is essential for the function of the oxidative phosphorylation system and cellular homeostasis. Mitochondrial DNA is particularly susceptible to aging-related oxidative stress due to the lack of a histone coat. Disturbances in mitochondrial DNA may contribute to functional decline during the aging process and in neurodegenerative diseases, leading to further impairment of mitochondrial DNA and initiating a vicious cycle. To date, it remains unclear how disturbed mitochondrial DNA is involved in the etiology of pathological aging and neurodegenerative diseases. The purpose of this review is to clarify the crucial roles of mitochondrial DNA homeostasis in the pathogenesis of neurodegenerative diseases. Mitochondrial DNA is distributed within nucleoids and is then transcribed into polycistronic mitochondrial DNA molecules within the mitochondrial granule region. Within the ultrastructure of the mitochondrial nucleoid and granule, a group of essential mitochondrial proteins involved in DNA replication, DNA transcription, RNA translation, RNA surveillance, and RNA degradation plays a crucial role in maintaining mitochondrial structure, genome integrity, and mitochondrial DNA processing. The uniparentally inherited mitochondrial DNA undergoes heritable polyploid variations, which include homoplasmy and heteroplasmy. Accumulating mitochondrial DNA alterations, such as deletions, point mutations, and methylations, occur during the pathogenic processes of neurodegenerative diseases. The increased mitochondrial DNA alterations can be propagated by the rise of deleterious heteroplasmy in neurodegenerative diseases, ultimately resulting in impairment to the oxidative phosphorylation system, biogenesis defects, and cellular metabolic dysfunction. Therefore, developing appropriate gene editing tools to rectify aberrant alterations in mitochondrial DNA and targeting the key proteins involved in maintaining mitochondrial DNA homeostasis can be considered promising therapeutic strategies for neurodegenerative diseases. Although therapeutic strategies targeting mitochondrial DNA in diseases show great potential, challenges related to efficacy and safety require a better understanding of the mechanisms underlying mitochondrial DNA alterations in aging and neurodegenerative diseases.

**Facts**

• The review highlights the crucial roles of mitochondrial DNA homeostasis in the occurrence and progression of various neurodegenerative diseases.

• Novel intervention strategies, including gene editing to address mtDNA mutations, deletions, and methylation changes, as well as mitochondrial transplantation and the use of antioxidants, have demonstrated potential in animal models for improving mitochondrial function and slowing down disease progression.

• The ongoing advancement in technologies such as single-cell sequencing and high-depth sequencing has uncovered the relationships between mitochondrial DNA heterogeneity, threshold effects, and haplotypes in relation to disease risk, thereby laying the groundwork for personalized precision treatment

**Open questions**

• How do mitochondrial DNA copy number and mutations transition from cellular levels to those in tissues, organs, and even behavioral phenotypes across various types and stages of neurodegenerative diseases?

• Can mitochondrial DNA haplotypes and heterogeneity serve as stable clinical biomarkers for predicting disease risk and treatment response?

• How can we improve the delivery efficiency and clinical feasibility of mitochondrial DNA-targeted therapies, such as gene editing and mitochondrial transplantation, in the central nervous system while ensuring safety?

## Introduction

Mitochondria are ubiquitous organelles with an array of functions, from cellular energy metabolism, signaling sensing, and immune responses to organismal regulation (Aubry et al., 2025; Cicali et al., 2025). Mitochondria provide the main source of cellular energy through the electron transport chain to generate ATP via the oxidative phosphorylation (OXPHOS) system (Paggio et al., 2019; Wescott et al., 2019; Gezen-Ak et al., 2020). Unlike other organelles, mitochondria contain their own mitochondrial DNA (mtDNA), which encodes 37 genes, including 13 that are essential for the respiratory apparatus, along with 2 rRNA genes and 22 tRNA species. The small genome contains around 16.5 kbp in humans that could extremely affect OXPHOS function (Mercer et al., 2011).

Unlike nuclear DNA, mtDNA is characterized by the polycistronic form and lack of introns (Anderson et al., 1981). mtDNA and a group of proteins are located within mitochondrial nucleoids, and mtRNA and relevant proteins are distributed within mitochondrial granules region. These proteins are responsible for mtDNA replication, transcription, translation, and degradation. Great progress has been made in identifying the key components of the mtDNA replication, transcription, and translation machinery, indicating that precise regulation and a multistep process are essential for mtDNA homeostasis.

mtDNA undergoes higher mutation rates than nuclear DNA. Alterations in mtDNA can affect coding properties and lead to deleterious phenotypes in cellular metabolism. All organs of the body rely heavily on mitochondria for proper energy supply. Notably, the brain accounts for only about 2% of total body weight but consumes 20% of the body’s energy supply. Extensive observational data suggest that the genetic instability of mtDNA could severely impair respiratory chain function in neurons and other glial cells in the central nervous system (CNS). Unlike nuclear DNA, mtDNA is not packaged into nucleosomes with a histone coat, making it more vulnerable to oxidative stress. A previous study has reported that mtDNA is 10 to 100 times more susceptible to damage than nuclear DNA (Gupta et al., 2023). More subtle decreases in mtDNA copy numbers and accumulated somatic mtDNA mutations have been observed in somatic tissues during aging and the development of neurodegenerative diseases such as Alzheimer’s disease (AD), Parkinson’s disease (PD), Huntington’s disease (HD), amyotrophic lateral sclerosis (ALS), frontotemporal lobe degeneration (FTLD), and multiple sclerosis (MS) (Kukreja et al., 2014; Bennett and Keeney, 2020; Shang et al., 2023).

**Figure 1:**
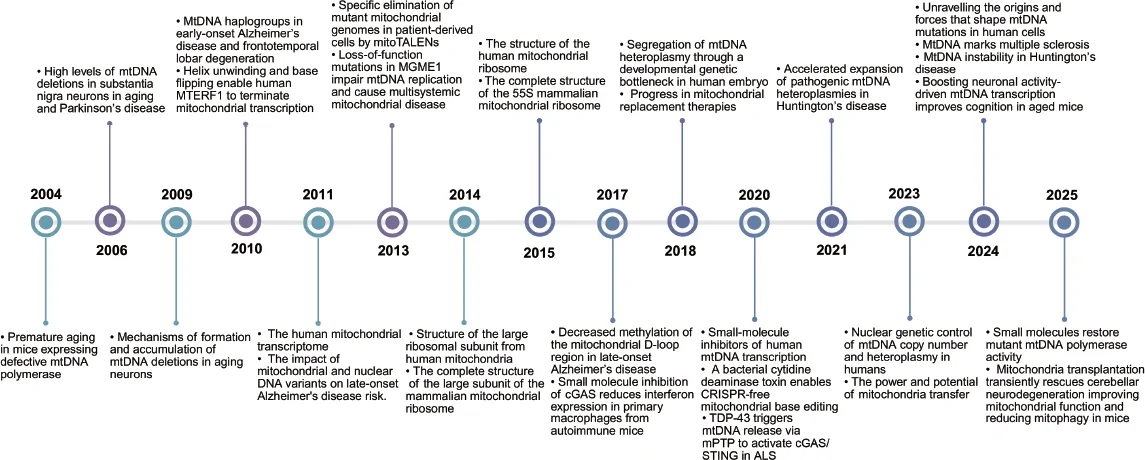
Timeline and milestone events in the study of mitochondria and mitochondrial DNA homeostasis in neurodegenerative diseases. ALS: Amyotrophic lateral sclerosis; cGAS: cyclic GMP-AMP synthase; CRISPR: clustered regularly interspaced short palindromic repeats; D-loop: displacement loop; MGME1: mitochondrial genome maintenance exonuclease 1; mitoTALENs: mitochondrial transcription activator-like effector nucleases; mPTP: mitochondrial permeability transition pore; mtDNA: mitochondrial DNA; STING: stimulator of interferon genes; TDP-43: TAR DNA-binding protein 43.

Neurodegenerative diseases can be sporadic or inherited disorders with progressive loss of neurons and irreversible neuronal injury (Liang et al., 2025; Xia et al., 2025). Neurodegenerative diseases are induced by the accumulated damage in response to various stressors during the brain aging process. The neurons in the brain, which are high energy demanding, are especially vulnerable to increased damaged mtDNA. Swerdlow et al. (2020) described individual mtDNA haplogroups on the risks of pathological aging and the development of neurodegenerative diseases. Although the mtDNA is exclusively inherited in maternally transmitted pattern (Alexeyev and Bai, 2025), the increased abundance of mtDNA mutations can be spread by replication and increase the level of deleterious heteroplamy in neurodegenerative diseases, resulting in mitochondrial dysfunction and further leading to synaptic impairment and neuronal injury (Bi et al., 2023; Gupta et al., 2023; Dua and Badrinarayanan, 2024). Targeted modulation of the maintenance of mtDNA homeostasis may restore neuronal impairment and promote neural regeneration. Given the important role of mtDNA, it is important to understand the involvement of somatic mtDNA alterations in the pathogenesis of aging and neurodegeneration (**Figure 1**).

In this review, we aim to provide a comprehensive overview of mtDNA alterations in aging and neurodegenerative diseases, as well as their regulatory mechanisms in pathogenesis. We describe in detail the processing of mtDNA and the relevant regulators involved in mtDNA replication, mtDNA transcription, mtRNA translation, mtRNA surveillance, and mtRNA degradation. Furthermore, we explore the possibilities of targeting mtDNA to treat neurodegenerative diseases and discuss potential therapeutic strategies and limitations based on the characteristics of genetic alterations in aging and neurodegenerative diseases, offering references and novel insights for clinical application.

## Search Strategy

An online search of the literature describing basic and clinical research on PubMed from 1956 to 2025 was performed using the following keywords: mitochondrial genome AND neurodegenerative diseases; mtDNA AND neurodegenerative diseases; mtDNA AND aging; mtDNA AND Alzheimer’s disease; mtDNA AND Parkinson’s disease; mtDNA AND Huntington’s disease; mtDNA AND amyotrophic lateral sclerosis; mtDNA AND frontotemporal lobe degeneration; mtDNA AND multiple sclerosis; and mtDNA AND therapeutic. The results were further screened by title and abstract. Studies that examined the relationship between mtDNA and neurodegenerative diseases were included, while articles discussing mtDNA that were not related to neurodegenerative diseases were excluded.

## Mitochondria and Mitochondrial Genomes

### Intra-mitochondrial structure: Mitochondrial DNA nucleoids and granules

**Figure 2:**
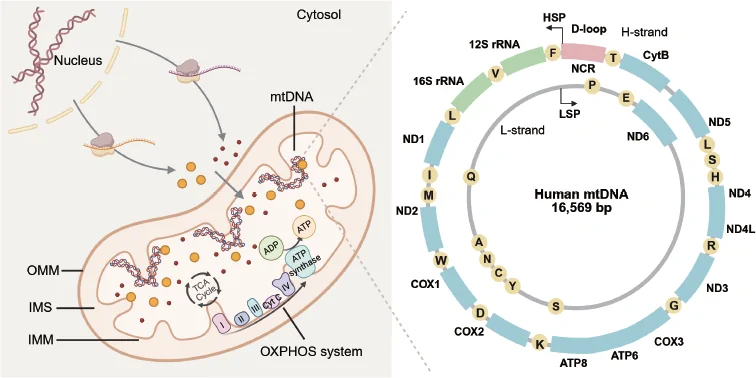
Mitochondrial structure (left) and organization of the human mitochondrial genome (right). A double-strand polycistronic mtDNA molecule including 37 genes encoding the subunits of the OXPHOS system. Created with BioRender.com. D-loop: Displacement loop; HSP: heavy strand promoter; IMM: inner mitochondrial membrane; IMS: intermembrane space; LSP: light strand promoter; mtDNA: mitochondrial RNA; NCR: noncoding region; OMM: outer mitochondrial membrane; OXPHOS: oxidative phosphorylation.

Mitochondria can vary in shape from ovoid to rod-shaped, displaying no fixed morphology; they can appear tubular, elongated, fragmented, or even form a highly connected reticular network. Mitochondria are specialized organelles characterized by a double-membrane arrangement that divides them into four compartments: the outer mitochondrial membrane, inner mitochondrial membrane, intermembrane space, and matrix (Grba and Hirst, 2020; Zhang et al., 2021; **Figure 2**). The outer mitochondrial membrane is porous and allows ions and uncharged molecules to freely traverse through voltage-dependent anion channels, thereby separating the mitochondria as autonomous organelles from the cytoplasm (Callegari et al., 2025). Due to its porous structure, there is no membrane potential across the outer mitochondrial membrane. The inner mitochondrial membrane is folded into cristae, where super-complexes of the respiratory chain and ATP synthesis machinery are embedded, creating a tight diffusion barrier against ions and macromolecules (Kuhlbrandt, 2015). Larger proteins must be transported by specific membrane translocases that exhibit selectivity for particular ions or molecules. The convoluted and adaptive alterations in the architecture of cristae are correlated with aging and various diseases, depending on cell type and stress (Caron and Bertolin, 2024). The highly folded cristae create two aqueous compartments: the intermembrane space and the matrix. The matrix contains mtDNA, RNA, and proteins. The mtDNA is packaged into mtDNA-protein complexes known as nucleoids, which serve as organizing bodies for mtDNA replication, transcription, translation, protein biosynthesis, and enzymatic reactions.

Mitochondria, as semi-autonomous organelles, contain their own double-stranded genome, mtDNA, which is transmitted strictly through maternal inheritance but lacks histones and other machinery for genome compaction (Papadea and Larsson, 2024). The mitochondrial nucleoid, a highly compact spherical sub-organelle within mitochondria, serves as a platform for the organization of mitochondrial genomes. Nucleoids were first described in 1937 by Piekarski to define the membrane-lacking structure of the bacterial chromosome in eukaryotes (Robinow and Kellenberger, 1994). The nucleoid has a diameter of approximately 100 nm, allowing it to fit within the 10 μm tubules of the mitochondrial reticular network, which is regulated by fission and fusion due to the highly dynamic nature of mitochondria (Jezek and Dlaskova, 2019). The initiation of mtDNA replication and transcription requires several proteins in the core region of mitochondrial nucleoids, including mitochondrial transcription factor A (TFAM), mitochondrial transcription factors B1 and B2 (TFB1M/TFB2M), mitochondrial transcription elongation factor (TEFM), mitochondrial single-stranded DNA-binding protein (mtSSB), mitochondrial polymerase γ (POLG), mitochondrial RNA polymerase (POLRMT), and TWINKLE helicase (Ghrir et al., 1991; Gleyzer et al., 2005; Hensen et al., 2019; Xu et al., 2023; Huh et al., 2024). The mtDNA undergoes phase separation through the interaction with TFAM, which facilitates mitochondrial nucleoid self-assembly and is crucial for promoting mtDNA transcription (Isaac et al., 2024). Other processes, such as the organization of respiratory chain supercomplexes, endoplasmic reticulum-mitochondria complexes, mitochondrial ribosomes termed mitochondrial organization of gene expression complexes, and mitochondrial membrane supercomplexes involved in protein trafficking, must be organized into higher-order assemblies (Schagger and Pfeiffer, 2000; Kornmann and Walter, 2010; Kehrein et al., 2015).

The mtDNA within nucleoids is transcribed into single-stranded polycistronic mtRNA molecules. In addition to mitochondrial nucleoids, another membraneless structure containing mtRNA within mitochondria has been identified as mitochondrial RNA granules (MRGs). The MRGs are approximately 90–130 nm in diameter and consist of sub-compartmentalized liquid condensates (Landoni et al., 2024). They are in contact with the inner mitochondrial membrane and are randomly distributed along the mitochondrial reticular network, undergoing dramatic dynamic molecular interchange through fusion, fission, and branching (Rey et al., 2020). The MRGs are identified as sites for the spatiotemporal regulation of mtRNA processing and maturation, mitochondrial translation, and the biogenesis of mitochondrial ribosomes. The ultrastructure of the nucleoid and granules highlights the importance of understanding the regulatory relationship between mitochondrial nucleoids or granules and mitochondrial genomes. This understanding provides a basis for elucidating the pathological processes associated with mitochondria and offers insights into novel therapeutic regimens for treating mitochondria-associated diseases in clinical settings.

### General characteristics of mitochondrial DNA

The number of mtDNA copies varies greatly across different species and cell types, as well as in various diseases such as mitochondrial disorders, aging, cancer, and neurodegenerative diseases. In mammals, mature oocytes contain more than 15,000 copies of mtDNA, while sperm contain only approximately 100 copies (Wai et al., 2010). The transcription of mtDNA is initiated from the non-coding region (NCR), which spans around 1000 bp between the tRNA genes for proline and phenylalanine. The NCR includes the heavy-strand promoter (HSP), the light-strand promoter (LSP), and the origin of heavy-strand replication (OH), and it incorporates a linear third strand of 7S DNA, forming a stable mitochondrial displacement loop (D-loop) structure (Nicholls and Minczuk, 2014). Human mtDNA is characterized as an intronless circular DNA molecule of 16,569 bp, encoding 13 protein subunits of the OXPHOS system, 2 rRNAs (12S and 16S), and 22 tRNAs (Anderson et al., 1981; Mercer et al., 2011). Among these genes, 12 OXPHOS subunits, 2 rRNAs, and 14 tRNAs are transcribed from the heavy strand, initiated from HSP. The genes coding for subunit 6 of NADH dehydrogenase (ND6), 8 tRNAs, and a group of non-coding RNAs are transcribed from the light strand under the control of LSP (Jedynak-Slyvka et al., 2021; **Figure 2**). The processing of the mitochondrial genome is highly complex and involves multiple levels of regulation, including mtDNA replication, transcription, translation, and degradation. The integrity of the mitochondrial genome is essential for cellular homeostasis. Disturbances in mtDNA replication and the incremental accumulation of mtDNA mutations are closely linked to the aging process, cancer, and neurodegenerative diseases.

### Mitochondrial DNA replication

In mammals, mtDNA replication occurs in a consecutive manner without the formation of Okazaki fragments. mtDNA contains dedicated replication origins on two strands: the heavy-strand origin (O_H_) and the light-strand origin (O_L_). mtDNA replication initiates from O_H_, and during the first phase of replication, heavy-strand (H-strand) DNA synthesis occurs without simultaneous synthesis of the complementary light strand (L-strand). The minimal mtDNA replisome machinery is composed of numerous factors, including POLG, the TWINKLE DNA helicase, mtSSB, and POLRMT (Falkenberg and Larsson, 2009). POLG serves as the core of the mtDNA replisome and is the only replicative polymerase in the form of a heterotrimeric complex in mitochondria. POLG consists of a catalytic POLG A subunit and two accessory POLG B subunits. POLG A belongs to the family A group of DNA polymerases and has a molecular mass of 140 kDa. It contains a 3′ to 5′ exonuclease domain with exonuclease activity, which ensures efficient proofreading during DNA synthesis, with an error frequency of less than 1 × 10^6^ per base (Longley et al., 2001). The accessory subunit POLG B exhibits structural homology with class IIa aminoacyl-tRNA synthetases and has a molecular mass of 55 kDa (Carrodeguas et al., 2001; Fan et al., 2006). POLG B is a dimer, with distinct functions for each monomer. One subunit stabilizes the binding affinity with double-stranded DNA (dsDNA) substrates, while the other monomer stimulates the polymerization rate by interacting with POLG A during elongation. During mtDNA replication, POLG cannot use dsDNA as a template and requires the assistance of the DNA helicase TWINKLE for DNA synthesis and unwinding of the mtDNA duplex, which occurs in a 5′ to 3′ direction. The DNA helicase TWINKLE is part of Superfamily 4 (SF4) and is homologous to the bacteriophage T7 replicative helicase, with 46% sequence similarity and 15% identity (Holmlund et al., 2009). Like other SF4 helicases, TWINKLE undergoes dynamic oligomeric state-shifting between heptameric arrangements (Peter and Falkenberg, 2020). TWINKLE is also the sole replicative helicase for mtDNA maintenance. Knockout of TWINKLE in mice is lethal to embryos and leads to severe deterioration of mitochondrial DNA homeostasis, including mtDNA depletion, impaired mtDNA expression, and respiratory chain deficiency. Mammalian TWINKLE is essential for mtDNA integrity in dopaminergic neurons. Mutant TWINKLE results in age-related mtDNA deletions and dopaminergic neuron degeneration, ultimately leading to PD (Song et al., 2012). The helicase activity of TWINKLE is enhanced by mtSSB, which helps stabilize extended segments of single-stranded DNA at the replication fork. mtSSB contains an N-terminal oligosaccharide/oligonucleotide-fold domain with a molecular weight of 16 kDa, and it is encoded by the SSBP1 gene. mtSSB not only binds to single-stranded DNA in a tetramer formation but also promotes mtDNA synthesis by enhancing the processivity and primer recognition of POLG (Riccio et al., 2024).

**Figure 3:**
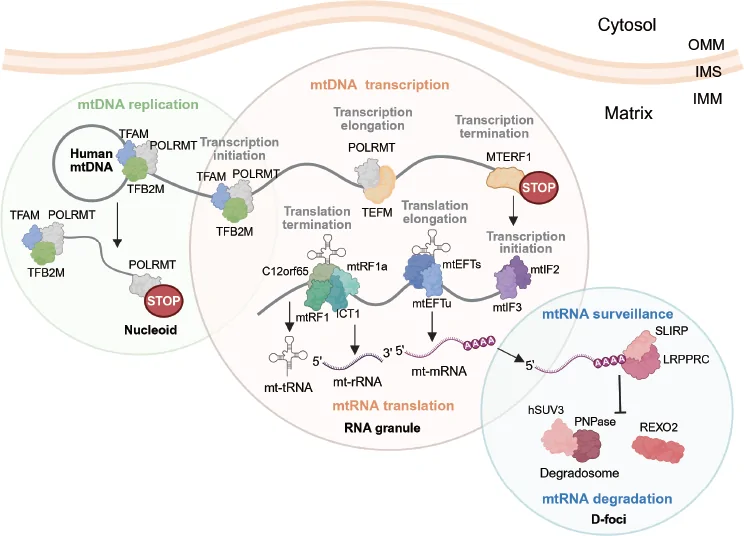
Schematic overview of mitochondrial gene expression, containing mtDNA replication, mtDNA transcription, mtRNA translation, mtRNA surveillance, and mtRNA degradation. mtDNA replication takes place in the mitochondrial nucleoid regions, which is represented by a green circle. mtDNA transcription and mtRNA translation occurs in the mitochondrial RNA granules, which is indicated by a yellow circle. mtRNA surveillance and degradation happen in the D-foci region, which is presented in a blue circle. Created with BioRender.com. LRPPRC: Leucine-rich pentatricopeptide repeat-containing protein; mtDNA: mitochondrial DNA; mtEFTs: mitochondrial elongation factor thermos stable; mtEFTu: mitochondrial elongation factor thermos unstable; MTERF1: mitochondrial transcription termination factor 1; mtIF2: mitochondrial initiation factor 2; mtIF3: mitochondrial initiation factor 3; mtRNA: mitochondrial RNA; PNPase: polynucleotide phosphorylase; POLRMT: mitochondrial RNA polymerase; REXO2: RNA exonuclease 2; SLIRP: stem-loop-interacting RNA-binding protein; TEFM: mitochondrial transcription elongator factor; TFAM: mitochondrial transcription factor A; TFB2M: mitochondrial transcription factors B2.

The initiation of mtDNA replication is strictly regulated, beginning at the O_H_ with primers synthesized by POLRMT. In the initial phase, the DNA helicase TWINKLE unwinds dsDNA ahead of the active POLG. The displaced single-stranded DNA is coated and stabilized by mtSSB. When the lagging-strand replication machinery reaches the O_L_, the parental H-strand in the O_L_ region folds into a stem-loop structure. This structure serves as an origin site for primer synthesis by POLRMT, which is then utilized by POLG to initiate L-strand DNA synthesis. Once POLRMT is replaced by POLG, the synthesis of both H- and L-strands continues until both strands are completed, resulting in two newly formed daughter molecules. After POLG has finished full-length mtDNA replication, the RNA primers used during mtDNA synthesis must be removed (**Figure 3**). The regulators involved in primer processing during mtDNA replication differ between O_H_ and O_L_. The degradation of the primer at O_L_ requires RNase H1. It has been demonstrated that RNase H1 cannot cleave the DNA strand; instead, it degrades the RNA portion of a chimeric strand that is annealed to DNA, leaving behind only 1–3 ribonucleotides at the junction between the RNA and DNA strands. The remaining ribonucleotides require additional nuclease activity, specifically from mitochondrial exonuclease G. Primer processing at O_H_ requires two nucleases: RNase H1 and mitochondrial genome maintenance exonuclease-1 (MGME1). During primer processing at O_H_, RNase H1 removes the RNA strand of the primer in the O_H_ region, while MGME1 is responsible for processing the DNA portion of the primer, including a nascent H-strand DNA segment approximately 100 nucleotides in length (Kornblum et al., 2013; Riccio et al., 2024).

### Mitochondrial DNA transcription

mtDNA replication and transcription are tightly coupled processes that involve three steps: initiation, elongation, and termination. The mitochondrial transcription machinery mainly includes POLRMT, TFAM, TFB2M, TEFM, and MTERF1. POLRMT is the most extensively researched mitochondrial transcription-associated factor of the mtDNA transcription apparatus (Park et al., 2007; Hillen et al., 2018). The single-subunit POLRMT partially resembles T7-like bacteriophage RNA polymerases (T7 RNAP) (Sousa et al., 1992). The immature POLRMT protein contains 1230 amino acids, of which 41 amino acids are cleaved off during mitochondrial import. This results in the production of a mature POLRMT protein consisting of 1189 amino acids. Although POLRMT is nuclear-encoded, it is incapable of transcribing any nuclear genes and is solely responsible for mtDNA transcription (Olahova et al., 2021). The catalytic domain of POLRMT is located within amino acids 657–1230 of the C-terminus, which adopts a right-hand fold with conserved fingers, palm, and thumb subdomains. The N-terminal domain, comprising amino acids 368–647 of POLRMT, is less conserved but is essential for promoter recognition and melting in T7 RNAP. Although POLRMT is an active processive enzyme such as RNA polymerase, it requires two transcription initiation factors, TFAM and TFB2M, to initiate mtDNA transcription. TFAM belongs to the high mobility group box (HMG-box) family and contains two tandem HMG-boxes, HMG1 and HMG2. TFAM performs multifunctional roles in mitochondria due to its critical contributions to mtDNA replication and transcription. The first role of TFAM is to package mtDNA into nucleoids through the interaction with local DNA without sequence specificity. There are approximately 1000 copies of TFAM per mtDNA molecule within the anomaly-shaped nucleoid. The second role of TFAM is to facilitate promoter recognition by POLRMT, allowing accurate initiation from HSP and LSP, which are mapped between bases –10 and –35 upstream of the transcription start site (TSS). This interaction results in DNA bending and recruits POLRMT to the TSS, likely by binding to the TFAM HMG2 domain and the lengthy tether helix of the POLRMT N-terminal domain (Posse et al., 2014; Hillen et al., 2017). In the absence of TFAM, there is low-level mtDNA transcription from mitochondrial promoters (Song et al., 2024b). The associations of TFAM and mtDNA copy number with different neurodegenerative diseases are complex. Genetic mutations and polymorphisms in TFAM are highly associated with the development of AD, PD, and HD. The cell type-specific ablation of TFAM could render neurons more susceptible to stress, further contributing to common neurodegenerative diseases (Kang et al., 2018; Song et al., 2024b). TFB2M is the second mammalian initiation factor required to recognize mitochondrial promoters and facilitate promoter unwinding. TFB2M adopts a methyltransferase fold and is highly similar to its paralog TFB1M, a highly conserved mitochondrial rRNA methyltransferase. *In vitro* research suggests that both TFB1M and TFB2M bind directly to POLRMT in a heterodimer conformation. In particular, TFB2M causes conformational alterations in POLRMT, enabling promoter opening and trapping the non-template strand in the open DNA region (Hillen et al., 2017).

After the initiation factors of mtDNA transcription are released during promoter escape, TEFM must be recruited for processive synthesis of mitochondrial transcription and for the transition to transcription elongation. TEFM forms a dimer in solution with a molecular weight of 38 kDa. Each monomer of the dimer is composed of two tandem helix-hairpin-helix domains that facilitate activities related to transcriptional elongation. Additionally, the dimeric conformation enables TEFM to complex with POLRMT near the RNA exit channel, forming a sliding clamp structure that promotes the processivity of POLRMT. Due to its localization near the RNA exit channel, TEFM can impede the formation of secondary structures in RNA, which may prevent premature termination of mtDNA transcription. TEFM is key to regulating the switch between mitochondrial replication and transcription, potentially connecting mtDNA transcription elongation and RNA processing. During the final phase of polycistronic transcription, each strand of mtDNA is terminated at specific sites. The L-strand termination site is located before the 3′ end of the rRNA gene cluster and is regulated by mitochondrial transcription termination factor 1 (MTERF1) (**Figure 3**). It has been identified that MTERF1 binds the mt-tRNALeu sequence and terminates specific transcription downstream of 16S mt-rRNA from HSP1 (Fernandez-Silva et al., 1997). Previous studies have shown that MTERF1 exhibits strong polarity in transcription termination from the LSP promoter but also guides polar replication fork pausing (Yakubovskaya et al., 2010; Shi et al., 2016). The knockout of MTERF1 acts as a roadblock to LSP transcription termination, but has minimal impact on the transcription of the H-strand (Terzioglu et al., 2013). The termination site of the H-strand is located within the termination-associated sequence (TAS), at the 3’ end of the NCR (Doda et al., 1981; Jemt et al., 2015). This region exhibits a stronger connection with the continuous cycle of mtDNA replication initiation and termination, generating a short DNA strand known as 7S DNA. This terminated replication product hybridizes to the L-strand, forming a triple-stranded structure termed the displacement loop (D-loop) (Nicholls and Minczuk, 2014). However, the underlying molecular mechanisms of H-strand transcription termination at this site have not yet been clearly elucidated.

### Mitochondrial RNA translation

After mtDNA transcription, mitochondria retain their own specific translation machinery required for mitochondrial protein synthesis. The mitochondrial translation machinery contains multiple ribosomes and translation factors. The application of cryo-electron microscopy (cryo-EM) in numerous studies has provided insights into the structural details and assembly process of mitoribosomes. In mammals, fully assembled and mature mitoribosomes are composed of 55 S particles with a molecular mass of 2.7 MDa. Each particle consists of a 39 S large subunit (LSU) and a 28 S small subunit (SSU). The mitochondrial LSU contains a structural tRNA (tRNA^Val^ or tRNA^Phe^), 16 S RNA, and 52 mitochondrial ribosomal proteins, while the mitochondrial SSU encompasses a 12 S RNA and 30 mitochondrial ribosomal proteins (Greber et al., 2014; Amunts et al., 2015). The functional integrity of the mitoribosome relies on the coordination between mitochondrial SSU and LSU assembly, which is mediated by protein-protein or protein-RNA interactions.

The cycle of mitoribosome function during mitochondrial translation consists of four stages: initiation, elongation, termination, and ribosome recycling. During the initiation phase, mRNAs bind to the mitochondrial SSU, and start codons are recognized by initiator tRNAs through Watson-Crick pairing. Two initiation factors, mitochondrial initiation factor 2 (mtIF2) and mtIF3, play key roles in mitochondrial translation. mtIF2 contains a specific mitochondrial domain insertion that compensates for the absence of mtIF1, preventing premature tRNA binding to the ribosomal A site during the formation of the initiation complex (Greber et al., 2014). The precise role of mtIF3 during translation initiation remains controversial. Studies suggest that mtIF3 facilitates translation initiation by promoting the interaction between mRNA and the SSU. Depletion of mtIF3 specifically affects the translation of bicistronic transcripts from both the H-strand and L-strand. The mitoribosome has multiple conserved tRNA-binding sites, including the aminoacyl (A) site, peptidyl (P) site, exit (E) site, and the GTPase-associated center. The GTPase-associated center contains the stalk base, the L7/L12 stalk, and the sarcin-ricin loop (Amunts et al., 2015; Greber et al., 2015). After the initiation complex is formed, mtIF2 binds to the sarcin-ricin loop and promotes GTP hydrolysis. Once the initiation factors dissociate from the assembled initiation complex, the transition to the translation elongation phase occurs. The elongation phase consists of three cycles: mRNA codon decoding by the specific aminoacyl-tRNA, peptide bond formation, and translocation of the mRNA-tRNA complex. Two elongation factors, mitochondrial elongation factor thermos stable (mtEFTs) and mitochondrial elongation factor thermos unstable (mtEFTu), are responsible for the selection and correct accommodation of the corresponding tRNA. The mtEFTu-GTP complex regulates the delivery of the aminoacyl-tRNA to the A site of the mitoribosome, facilitating the second elongation step, peptide bond formation. The peptidyl transferase reaction is catalyzed by the peptidyl transferase center (PTC) of the mitoribosome, which promotes the transfer of the nascent polypeptide from the P site to the α-amine of the A site tRNA (Brown et al., 2014; Greber et al., 2014). During the final phase of elongation, the deacetylated P site and A site tRNA, which carries the nascent polypeptide chain, move to the E and P sites, respectively. This process is regulated by mtEFG1 and mtEF4, which facilitate the translocation of the mitoribosomes toward the 5′ end of mRNA. During the termination stage, the stop codons in mitochondria exhibit significant differences from those in cytoplasmic translation. While UGA, UAG, and UAA are typical stop codons in the cytoplasm, UGA encodes tryptophan in human mitochondria (Barrell et al., 1979). Additionally, two alternative stop codons, AGA and AGG, which terminate translation of mtRNA COX1 and ND6, respectively, typically encode arginine in the cytoplasm (Anderson et al., 1981). Four translation termination factors are involved in this process: mtRF1, mtRF1a, ICT1, and C12ORF65. mtRF1 is responsible for terminating the translation of COX1 and ND6 and shares genetic associations with schizophrenia and neurodegenerative diseases (Chen et al., 2016; Nadler et al., 2022). mtRF1a performs multiple tasks during termination, including recognizing UAG and UAA stop codons and exhibiting peptidyl-tRNA hydrolase activity (Soleimanpour-Lichaei et al., 2007). ICT1 also recognizes the atypical stop codons AGG and AGA (Akabane et al., 2014). C12ORF65 binds to the RNA-binding protein MTERS1 to release the nascent peptide and tRNA from stalled mitoribosomes (Gopalakrishna et al., 2019).

### Mitochondrial RNA surveillance and degradation

The steady-state level of the mitochondrial genome is essential for maintaining homeostasis in living cells. mtRNA surveillance and degradation are pivotal for sustaining moderate turnover of mtRNA and ensuring quality control of the mitochondrial genome (Szczesny et al., 2010). The RNA-binding protein, leucine-rich pentatricopeptide repeat-containing protein (LRPPRC), is heavily involved in maintaining the stability of the mitochondrial genome and suppressing degradosome-mediated mtRNA decay (Pietras et al., 2018). LRPPRC may interact with Parkin, potentially contributing to the progression of PD. Additionally, specific minigene assays provide evidence that intronic LRPPRC variants can regulate pre-messenger RNA splicing, which is highly relevant to the pathogenesis of PD and other neurodegenerative diseases (Zhang et al., 2025). Once imported into mitochondria, LRPPRC binds to the stem-loop-interacting RNA-binding protein (SLIRP), forming the LRPPRC/SLIRP complex to properly load mt-mRNA into the mitoribosomal small subunit (**Figure 3**). The 130-kDa protein factor LRPPRC contains 33 PPR domains with a helix-turn-helix structure. These domains, arranged in tandem, constitute a superhelix groove that stabilizes mtRNA. Although the small 11-kDa protein cofactor SLIRP possesses an RNA recognition motif domain, it is not responsible for RNA binding or mt-mRNA polyadenylation. Silencing SLIRP induces the destabilization of OXPHOS, a lack of enzymatic activity, and a reduction in mt-mRNA levels, which suggests its role in maintaining mitochondrial genomic homeostasis (Singh et al., 2024).

The degradation of mtRNAs is mediated by a mitochondrial degradosome, a complex consisting of the ATP-dependent RNA helicase SUV3 and polynucleotide phosphorylase (PNPase, also known as PNPT1). SUV3 is a homodimeric helicase that catalyzes the unwinding of RNA duplexes, preferentially unwinding RNA-RNA, DNA-DNA, and DNA-RNA duplexes (Jarrige et al., 2001; Walter et al., 2002; Szczesny et al., 2010; Li et al., 2025). The heteroduplex structure formed by nascent RNA-mtDNA hybrids leads to the creation of R-loops (Silva et al., 2018). The ribonuclease PNPase catalyzes the processive phosphorolytic degradation of RNA from the 3′ to 5′ end. A portion of PNPase is distributed within the mitochondrial intermembrane space, where SUV3 is absent. This pool of PNPase may help block the leakage of mtDNA from mitochondria to the cytosol, further preventing inflammation (Dhir et al., 2018). *In vivo*, the mitochondrial degradosome forms in distinct regions within mitochondria known as D-foci, where mtRNA degradation occurs. Knockdown of PNPase or SUV3 leads to a significant accumulation of mitochondrial antisense transcripts (Rubalcava-Gracia et al., 2024; Singh et al., 2024). Dysregulation of the degradosome results in the accumulation of R-loop structures in mtDNA, leading to instability of the mitochondrial genome (Silva et al., 2018). The end products of PNPase/SUV3 complex-mediated mtRNA degradation are short oligoribonucleotides. The RNA exonuclease REXO2 has been identified as the enzyme that removes these short RNA species (Bruni et al., 2013; Nicholls et al., 2019; Szewczyk et al., 2020). REXO2 exhibits strong specificity for oligonuclease activity toward short RNA substrates (**Figure 3**). Knockdown of REXO2 results in the accumulation of short oligoribonucleotides and double-stranded RNAs, while knockout of REXO2 disrupts mtRNA processing and mitochondrial homeostasis (Szewczyk et al., 2020). To date, many underlying mechanisms of mtRNA degradation remain to be elucidated, and the stability of the mitochondrial genome has not been fully clarified. However, the findings obtained thus far could inspire new perspectives on mtRNA degradation and elucidate the significant mechanisms involved in mitochondrial genomic homeostasis.

### Mitochondrial genomic homeostasis in aging

Aging is an intricate biological process that involves chronic deterioration in multiple organs and cells within an individual. Due to the similarities in the aging process, mitochondrial genomes undergo coordinated variations. Various environmental insults during aging, including stress, malnutrition, UV irradiation, and chemical toxicants, have adaptive effects on mitochondrial genomes, which may lead to transgenerational programming of mitochondrial dysfunction inheritance. There is a complex interplay between mitochondrial homeostasis and neuronal activity during aging (Li et al., 2024).

The instability of mitochondrial homeostasis manifests as alterations in mtDNA, including mtDNA mutations, mtDNA turnover, and mtDNA release-derived senescence-associated secretory phenotype (SASP) (Victorelli et al., 2023). Studies using human clinical data have shown the accumulation of mtDNA mutations in multiple organs of elderly subjects, including the CNS, skeletal muscle, heart, hepatocytes, and ovary, indicating the importance of mtDNA stability (Corral-Debrinski et al., 1992; Fayet et al., 2002; Trifunovic et al., 2004; Niemann et al., 2017; Zhou et al., 2024). However, whether mtDNA mutations serve as a driving force throughout an individual’s lifetime and the subcellular mechanisms involved remain unknown.

The mitochondrial free radical theory of aging (MRFTA) has garnered attention for several decades. This theory proposes that mitochondrial reactive oxygen species (ROS) production increases and mitochondrial function declines during the aging process, accompanied by decreased activity of ROS-scavenging enzymes (Harman, 1956). Oxidative stress on mtDNA may result in strand breaks that lead to mutations in mtDNA. Furthermore, mtDNA mutations progressively accumulate with aging, resulting in impaired respiratory functions and disrupted mitochondrial turnover in living cells. The accumulation of mtDNA mutations leads to increased oxidative damage to DNA, RNA, proteins, and lipids through a vicious cycle (Fraga et al., 1990). This cycle causes a deficient supply of energy, abnormalities in the OXPHOS system, and increased susceptibility to cellular stress.

Additionally, mtDNA can be released into the cytoplasm, induced by ROS and other stress factors during aging, which can subsequently trigger activation of the cyclic GMP–AMP synthase (cGAS)–stimulator of interferon genes (STING) pathway. The cGAS-STING-dependent innate immune processes have been reported to induce neuropathological processes in AD, ALS, and FTLD, indicating that targeting this pathway may yield therapeutic benefits (Yu et al., 2020; Decout et al., 2021, 2023; Xie et al., 2023).

The aging-associated mtDNA mutations include point mutations, large-scale deletions, and tandem duplications (Wei, 1998). High-throughput next-generation sequencing techniques facilitate the sensitive, specific, and accurate determination of mitochondrial genomic variability in aging (Yang et al., 2022). Unlike nuclear DNA, which is protected by histones, mtDNA exhibits an approximately 10- to 20-fold higher sequence evolution rate and a 100- to 1000-fold higher mutation rate (Yang et al., 2022). The uniparentally transmitted mtDNA has the heritable polyploid nature of mitochondrial genetics, with both homoplasmy (the synchronous presence of identical mtDNA in the same individual) and heteroplasmy (the concurrence of two or more genotypes of mtDNA in a single cell). Because mtDNA is segregated along the maternal germline and in somatic tissues, variations in homoplasmy and heteroplasmy are dynamic in both mitotic and post-mitotic cells. Some mtDNA mutations affect the entire mitochondrial genome in a homoplasmic pattern, while others only affect some copies of mtDNA, resulting in heteroplasmic mutations. Previous studies have shown that considerable variations in heteroplasmy are associated with control region points, particularly D-loop polymorphisms, indicating a link between mitochondrial heteroplasmy and somatic aging (Ghivizzani et al., 1993; Lee et al., 2007). A study by Sondheimer et al. (2011) analyzed dynamic variations of mitochondrial heteroplasmy in 138 single-nucleotide polymorphisms (SNPs) within a cohort of 2491 subjects with valid ages (ranging from 0 to 60 years old). They found that around 30% of heteroplasmy mutations were inherited, while 70% of these could be eliminated in offspring. Moreover, these heteroplasmic mutations may undergo a loss of variation in some SNPs, leading to increased homoplasmic characteristics of mitochondrial genetics (Sondheimer et al., 2011). The specific inherited mtDNA variations related to mtDNA genetic groups are termed mtDNA haplogroups. In European populations, centenarians with haplogroup J (characterized by mutations in the genes for subunits of the OXPHOS complex I) are more closely associated with longevity than those ethnically matched younger controls. mtDNA mutations associated with haplogroup J lead cells to produce less ROS and ATP, placing them in an unstable situation. In Asian populations, haplogroup D (characterized by mutations affecting complex I) is overrepresented among Japanese centenarians (Sondheimer et al., 2011). A series of studies have indicated that the burden of mutations in mtDNA genes coding for subunits of OXPHOS complexes I, III, and V varies between subjects and control groups. The presence of mutations in complex I may be beneficial for longevity, whereas the co-existence of mutations in both complexes I and III or in both complexes I and V might reduce the likelihood of longevity (Sevini et al., 2014). There is a biochemical threshold for the percentage of mutant mtDNA that may have detectable effects on OXPHOS function and phenotype onset. The pathogenic mutations associated with this so-called “threshold effect” should reach at least 60% to 90% heteroplasmy (Nissanka and Moraes, 2020). It appears that mtDNA variability has diverse effects on lifespan, further influencing human health and longevity. However, haplogroup analysis of a larger number of samples across different geographical areas may help clarify the impact of somatic mtDNA mutations on human aging and longevity.

## Mitochondrial Genomic Homeostasis in Neurodegenerative Disease

### Mitochondrial DNA disturbances in the pathogenesis of Alzheimer’s disease

mtDNA mutations accumulate in the CNS during aging, increasing the prevalence of neurodegenerative diseases. AD is the most common neurodegenerative disorder, characterized by extracellular amyloid-β (Aβ) accumulation, intracellular neurofibrillary tangles with hyperphosphorylated tau, and the loss of cholinergic neurons in specific brain regions of the CNS (Rafii, 2016; Jessen et al., 2024). However, the initiation of AD pathology begins prior to the emergence of Aβ or tau (Scheltens et al., 2021). Alterations in mitochondrial genome-associated proteins and mtDNA levels have been found during the early initiation period in the brain tissues, plasma, serum, and cerebrospinal fluid (CSF) of patients with AD (Grazina et al., 2006; Risi et al., 2025; **Additional Table 1**). The role of TFAM in mtDNA homeostasis remains a complex but open question (Alexeyev, 2023). TFAM protein levels are associated with mtDNA copy numbers in neurodegenerative conditions. In the whole brains of patients with AD, mtDNA levels were decreased by 57%, harboring somatic mtDNA mutations in the NCR, accompanied by reduced OXPHOS function and synaptic function (Coskun et al., 2004). In the hippocampi of patients with AD, TFAM protein levels were reduced by 47%, along with decreased levels of peroxisome proliferator-activated receptor gamma coactivator 1-alpha (PGC-1α) and other proteins, including nuclear respiratory factors (NRF-1 and NRF-2), leading to impaired mitochondrial biosynthesis. Previous studies have shown that TFAM protein levels were reduced by 20% to 88% in the hippocampus of APP/PS1 mice compared with the control group, along with decreased expression of genes involved in insulin pathways and increased levels of prion protein (Pedros et al., 2014; Petrov et al., 2016).

**Additional Table 1:** mtDNA alterations in neurodegenerative disease

Disease	Alteration type	Observed change	Detection method	Result	Reference
AD	mtSNP	TFAM SNP rs1937 and rs2306604	PCR sequencing	TFAM haplotype containing rs1937 G and rs230660 A is a moderate risk factor for AD.	Gunther et al., 2004
AD	mtSNP	SHMOOSE	PCR sequencing	Closely associated with AD, neuroimaging, and transcriptomics.	Miller et al., 2023
AD	mtDNA mutation	*TAMM41* gene and *mtND4L* gene	Whole-exome sequencing	Significant associations in AD cases or mild cognitive impairment cases.	Zhang et al., 2022b
AD	mtDNA deletion	mtDNA A4977	Dilution-PCR	The levels of mtDNA A4977 in AD started high and declined to low levels by the age of 80 years.	Xu et al., 2019
AD	mtDNA point mutations	m.5633T→C, m.7476C→T, m.15812 G→A	PCR sequencing	Significantly upregulated in patients with AD.	Wei et al., 2017
AD	mtDNA methylation	Increased D-loop methylation	PCR sequencing	Higher methylation levels of D-loop region were found in individuals with mild cognitive impairment.	Stoccoro et al., 2020
AD	mtDNA methylation	Decreased D-loop methylation	PCR sequencing	Significantly decreased levels of mtDNA methylation in the D-loop region of patients with LOAD.	Stoccoro et al., 2020
PD	mtDNA deletion	Decreased mtDNA copy numbers	Real-time PCR	The dopaminergic substantia nigra neurons of patients with PD exhibited higher frequency of somatic mtDNA deletions.	Bury et al., 2017
PD	mtDNA point mutations	m. 85843G→A, m.10398A→G	PCR sequencing	The two mtDNA point mutations may contribute to the protective effects of haplogroup B5.	Liou et al., 2016
PD	mtDNA point mutations	mtND3 SNP m.10398A > G	PCR sequencing	mtND3 SNP is protective in PD.	Liou et al., 2016
HD	mtDNA methylation	Deacetylation of K135 residue of ATAD3A	PCR sequencing	It is required for Drpl-induced fragmentation in HD.	Zhao et al., 2019b
HD	mtDNA deletion	Higher mtDNA copy number	Real-time PCR	Increased level in the leukocytes of early-onset HD.	Jedrak et al., 2017
HD	mtDNA deletion	Decreased mtDNA copy number	Real-time PCR	Reduced in the peripheral leukocytes after onset of HD, suggesting a biphasic pattern in the development of HD.	Petersen et al., 2014
HD	mtDNA point mutations	m.5613T > C in mitochondrial tRNA^Ala^	PCR sequencing	Highly related to COX deficiency.	Filosto et al., 2016
HD	mtDNA point mutations	m.3243A > G	PCR sequencing	High proportion of m.3243A > G could deteriorate mitochondrial metabolic function and affect mitochondrial respiratory function.	Tranah et al., 2018
ALS	mtDNA deletion	Higher mtDNA copy number in SOD1 or C9orf72 carriers	PCR sequencing	SOD1 or C9orf72 mutations are accompanied with higher mtDNA copy number in ALS.	Stoccoro et al., 2020
ALS	mtDNA methylation	Decreased D-loop methylation	Bisultite pyrosequencing and real-time PCR	Opposite correlation with D-loop methylation levels in SOD1-mutant ALS.	Stoccoro et al., 2020
ALS	mtDNA methylation	Decreased D-loop methylation	MS-HRM and real-time PCR	Opposite correlation with D-loop methylation levels and mtDNA copy number	Zuo et al., 2021
FTLD	mtSNV	mtSNV burden accumulation	Real-time PCR	mtSNVs within C9orf72-mutant astroglia were more likely to induce mitochondrial dysfunction.	Nie et al., 2025
MS	mtDNA point mutations	mtDNA T4216C	Real-time PCR	mtDNA variation as contributor factor in susceptibility to the clinical progression of a subgroup of patients with MS	Andalib et al., 2016
MS	mtDNA point mutations	nt13708 G/A variation			Yu et al., 2008
MS	mtDNA deletion	Increased mtDNA concentration	Real-time PCR	mtDNA as an indicator of biomarker and treatment response	Pavlovic et al., 2024

AD: Alzheimer's disease; ALS: amyotrophic lateral sclerosis; ATAD3A: ATPase family AAA-domain-containing protein 3A; D-loop: displacement loop; Drp1: dynamin-related protein 1; FTLD: frontotemporal lobe degeneration; HD: Huntington's disease; MS: multiple sclerosis; mtSNV: mtDNA single-nucleotide variant; PCR: polymerase chain reaction; PD: Parkinson's disease; SNP: nucleotide polymorphisms; SOD1: superoxide dismutase 1; TAMM41: mitochondrial translocator assembly and maintenance 41 homolog; TFAM: mitochondrial transcription factor A.

Mitochondrial-wide association studies, the mitochondrial counterpart of genome-wide association studies, have robustly reported multiple TFAM SNPs associated with various phenotypes in patients with AD. Common types of TFAM mutations include haplotypes such as rs1937 and rs2306604 in the 10q21.1 locus (Gunther et al., 2004). The G allele in TFAM rs1937 moderately increases the risk of AD, while the C allele in this SNP acts as a protective factor against late-onset AD (Gunther et al., 2004). Studies using high-depth whole-exome mtDNA sequencing have identified a decreased mtDNA copy number and increased mtDNA heteroplasmy in patients with AD. Zhang et al. (2022b) analyzed 4220 mtDNA variations, including the mtND4L and nuclear-encoded mitochondrial gene TAMM41, from whole-exome sequencing data in individuals with AD and mild cognitive impairment. They observed significant associations of AD with mtND4L and TAMM41 in individuals with AD or mild cognitive impairment. Several types of mtDNA alterations have been identified in AD, including mtDNA deletion (mtDNA Δ4977) and mtDNA mutations (T→C/A→G and G→A/C→T transitions) (Xu et al., 2019). Additionally, mtDNA point mutations are significantly upregulated in patients with AD; notable examples include m.5633T→C, m.7476C→T, m.15812 G→A, a tRNA gene variant at nucleotide pair 5075, and a tRNA gene variant at nucleotide pair 3646 (Hutchin et al., 1997; Chagnon et al., 1999; Qiu et al., 2001; Wei et al., 2017). In addition to mtDNA mutations and deletions, mtDNA methylation has emerged as another detrimental factor affecting mitochondrial function in AD. In an APP/PS1 mouse model, demethylation of the D-loop region was reduced (Xu et al., 2019). This phenotype has also been identified in patients with late-onset AD (LOAD). Higher methylation levels of the D-loop region were found in individuals with mild cognitive impairment, whereas Stoccoro et al. (2017, 2022) detected significantly decreased levels of mtDNA methylation in the D-loop region of LOAD patients. Brendan et al. (2023) detected a mitochondrial SNP (mtSNP) within a small open reading frame named small human mitochondrial ORF over serine tRNA (SHMOOSE), which is closely associated with AD, neuroimaging, and transcriptomics. Additionally, SHMOOSE levels in CSF correlate with age, tau levels in CSF, and brain white matter microstructure, although no significant correlation was found between SHMOOSE mtSNP and Aβ_1–42_ levels in CSF (Miller et al., 2023). Krüger et al. (2010) screened 194 subjects with early-onset AD and FTLD for the analysis of mtDNA haplogroups (H, V, U, K, J, T, I, W, X) along with two common point mutations (m.3243A→G, m.8344A→G), five POLG1 mutations (T2511, A467T, P587L, W748S, and Y955C), as well as mutations in progressive external ophthalmoplegia and adenine nucleotide translocator. Although they found subtle associations between these mtDNA mutations and AD or FTLD, their analysis revealed a significant phylogenetic correlation between mtDNA haplogroup cluster IWX and FTLD, indicating haplogroup-specific differences in the intensity of selection for mtDNA mutations in aging and neurodegenerative diseases (Kruger et al., 2010). Certain mtDNA haplogroups have been associated with increased or decreased risk of AD. For example, van der Walt et al. (2004) found that men with haplogroup U had an increased risk of AD, while Bi et al. (2015) reported that haplogroup B5a is commonly identified in AD cases. Tranah et al. (2014) found that participants with haplogroup L1 have an increased risk of developing dementia, with lower plasma Aβ_1–42_ levels. However, the downstream effects of these findings could not be adequately assessed against other risk factors for AD. Tranah et al. (2014) also identified that haplogroup L3, associated with the p.V1931 substitution in ND2, carries an increased risk of AD with significantly higher Aβ_1–42_ levels. Furthermore, haplogroups K and U may neutralize the effects of APOE4 allele (Carrieri et al., 2001; Kruger et al., 2010), but H, HV, and HV5 have synergistic effects with APOE4 allele (Maruszak et al., 2009, 2011; Santoro et al., 2010). mtDNA mutations and haplogroups can amplify the phenotypic manifestation in cascades during the AD pathological process, indicating their potential to be further translationally applied in clinical prediction.

### Mitochondrial DNA disturbances in the pathogenesis of Parkinson’s disease

PD is a common neurodegenerative disorder characterized by the progressive loss of dopaminergic neurons in the substantia nigra of the midbrain. The etiology of PD is complex, resulting from an interplay of genetic and environmental factors (Muhammed et al., 2015; Barnett, 2016; He et al., 2024b; Morris et al., 2024). Mitochondrial dysfunction plays a crucial role in the pathogenesis of PD, arising from impaired mitochondrial biogenesis, disturbances in the mitochondrial genome, and compromised OXPHOS function. Since mtDNA encodes components of the OXPHOS complex, an adequate amount of mtDNA is essential for the formation of OXPHOS subunits and the supply of neuronal energy. Current studies have reported lower mtDNA copy numbers in patients with PD (**Additional Table 1**). In particular, the dopaminergic neurons of the substantia nigra in patients with PD exhibit a higher frequency of somatic mtDNA deletions, suggesting that mtDNA homeostasis is disturbed in PD (Dolle et al., 2016). However, the phenotypic manifestations of mtDNA deletion and mtDNA copy number vary among different cell types and brain regions in individuals with PD. Bury et al. (2017) reported increased levels of mtDNA copy number and deletions in pedunculopontine cholinergic neurons of PD. Emilie et al. (2023) found that the lack of neuronal type I interferon (IFN) resulted in oxidation and deletion of mtDNA. Injection of the damaged mtDNA into the brains of mice could induce PD-dementia-like behaviors (Tresse et al., 2023; Wood, 2023). Further studies are needed to elucidate the regulatory mechanisms of mtDNA alterations in different cell types.

According to studies of cybrid cell lines, familial PD is primarily caused by nuclear genomic mutations, whereas sporadic PD is linked to variations in mtDNA (Swerdlow, 2012). The transmitochondrial cybrid cell line serves as a convenient model for investigating the pathophysiological consequences of mtDNA mutations in the context of PD. Cybrids are created by fusing an enucleated donor cell derived from the cytoplasts of PD patients with immortalized cell lines that lack mtDNA (King and Attardi, 1989). Numerous clinical data have validated that the risk of PD is significantly correlated with mtDNA haplogroups. Cybrids from PD cases with mtDNA haplogroup B5 exhibited a decreased risk of PD, showing higher mitochondrial membrane potential, lower autophagy gene expression levels, and reduced ROS production compared with control group. Two mtDNA point mutations (m. 85843G→A, m.10398A→G) may contribute to the protective effects of haplogroup B5 (Liou et al., 2016). Data from a large sample of Australian PD cases indicated that haplogroups J and K are associated with a decreased risk of developing PD compared with population-based controls (Mehta et al., 2009). Similarly, data from European mtDNA haplogroups demonstrated a significant downregulation in the risk of PD for haplogroups J and K compared with carriers of haplogroup H (van der Walt et al., 2003). Huerta et al. identified the mtND3 SNP m.10398A>G as being protective in PD. Consequently, mtDNA alterations may be considered potential hallmarks of neurodegenerative diseases.

### Mitochondrial DNA disturbances in the pathogenesis of Huntington’s disease

HD is a dominantly inherited neurodegenerative disorder characterized by cognitive decline and movement disorders. HD is associated with a genetic mutation in the Huntingtin protein (mHTT) (Bates, 2003). The mHTT protein results from a CAG trinucleotide repeat expansion mutation, with 36–121 repeats (normal is less than 35 repeats), leading to an elongated glutamine tract (poly-Q) (Elena-Real et al., 2023; Kiani, 2023; Llamas et al., 2023; Sun et al., 2024). The wild-type HTT is expressed in multiple tissues, but it shows the highest levels in the brain. Expression of mHTT can impair mitochondrial quality control and accelerate mitochondrial aging, which contributes to progressive neuronal defects and disrupts brain development. Somatic nuclear HTT CAG instability can easily lead to the accumulation of mHTT fragments, resulting in abnormal mitochondrial dynamics and mitophagy (Ray, 2011). The N-terminal, but not full-length, mHTT fragments are crucial for mitochondrial fission dynamics and mitophagy. Elevated levels of N-terminal mHTT can lead to detrimental mtDNA mutations and mtDNA instability (Orr et al., 2008; Neueder et al., 2024). Several mitochondrial proteins involved in mtDNA maintenance, including ATPase family AAA-domain-containing protein 3A (ATAD3A), silent information regulator sirtuin 1 (SIRT1), PGC-1, and TFAM, play significant roles in the pathogenesis of HD (Orr et al., 2008; Jeong et al., 2011). Proteomic analysis has identified that deacetylation of the K135 residue of ATAD3A is required for dynamin-related protein 1 (Drp1)-induced mitochondrial fragmentation in HD (Zhao et al., 2019b; **Additional Table 1**). The expression levels of SIRT1 and PGC-1 are decreased in HD patients with HD compared with the control group. Ultra-deep mtDNA sequencing has revealed accumulated mtDNA mutations related to OXPHOS function in somatic tissues of patients with HD. The mtDNA/nDNA copy number was significantly increased in the leukocytes of patients with HD. Notably, women with HD exhibited higher mtDNA levels in leukocytes than men with HD, suggesting that women with HD experience faster rates of disease progression than their male counterparts (Jedrak et al., 2017). However, another longitudinal study found that mtDNA copy number was reduced in peripheral leukocytes after the onset of HD, indicating that the alteration of mtDNA/nDNA follows a biphasic pattern in the development of HD (Petersen et al., 2014).

The mtDNA heterogeneity in the lymphocytes of patients with HD is also associated with the development of HD pathogenesis. Molecular analysis identified a novel m.5613 T > C heteroplasmic mutation in a muscle biopsy from a patient with HD, which is a maternally inherited mtDNA point mutation in the mitochondrial tRNAAla gene. The m.5613 T > C heteroplasmic mutation is considered to carry pathogenic risk. The heteroplasmic mutation load of m.5613 T > C is highly related to COX deficiency, as demonstrated by single-fiber PCR analysis (Filosto et al., 2016). Another mtDNA heterogeneity was identified at m.3244 in the mitochondrial tRNALeu gene. At this site, m.3243A > G or A > T point mutations appear four times more frequently in patients with HD compared with controls. The m.3243A > G mutation is the most common mtDNA mutation and is a significant risk factor for multiple diseases, including lactic acidosis, mitochondrial encephalopathy, and stroke-like episodes (Goto et al., 1990; Grady et al., 2018; Tranah et al., 2018). In HD, the high proportion of m.3243A > G may impair mitochondrial metabolic function and affect mitochondrial respiratory function (Picard et al., 2014; Kopinski et al., 2019). This accelerated accumulation of pathogenic mtDNA mutations in patients with HD provides mechanistic insight into mtDNA instability in the development of HD.

### Mitochondrial DNA disturbances in the pathogenesis of amyotrophic lateral sclerosis

ALS is a fatal neurodegenerative disease characterized by the degeneration of both upper and lower motor neurons, leading to muscle weakness and ultimately paralysis (Brown and Al-Chalabi, 2017; Feldman et al., 2022). The mechanisms underlying the pathogenesis of ALS remain poorly understood. While a subset of patients with ALS develops familial forms due to genetic mutations that affect neuronal function, the genetic risk factors for ALS primarily involve specific pathogenic variants, such as superoxide dismutase 1 (SOD1), fused in sarcoma (FUS), C9orf72, ACSL5, and TDP43 (Chia et al., 2018; Nakamura et al., 2020). In the molecular pathomechanisms of ALS, SOD1 plays a pivotal role in regulating mitochondrial function and oxidative stress. FUS, C9orf72, and TDP43 are associated with DNA repair and RNA metabolism in ALS, highlighting their essential roles in maintaining mtDNA stability. Genetic screening has found that SOD1 or C9orf72 mutations are linked to a higher mtDNA copy number in patients with ALS compared with control subjects. Both SOD1 carriers and patients with sporadic ALS exhibit decreased D-loop methylation levels compared with control group. Furthermore, the mtDNA copy number shows an inverse correlation with D-loop methylation levels in patients with SOD1-mutant ALS. Additionally, abnormal mtDNA methylation levels in the D-loop region and 16S rRNA gene have been reported in the skeletal muscle and spinal cord of SOD1 transgenic ALS mice (Stoccoro et al., 2020). It appears that demethylation of the D-loop region serves as a compensatory mechanism for mtDNA copy number in patients with SOD1-mutant ALS (**Additional Table 1**). TDP43 is a nuclear DNA/RNA-binding protein with genetic mutations found in 4% of patients with familial ALS. TDP43 aggregation in neuronal cells is triggered by oxidative stress (Zuo et al., 2021). This aggregation occurs in both the cytoplasm and mitochondria. Mislocalized TDP43 can disrupt mitochondrial homeostasis and activate hyperinflammatory response pathways, including those related to nuclear factor κB (NF-κB) cytokines, and IFN signaling. Yu et al. (2020) indicated that TDP43-driven inflammation depends on the cGAS/STING pathway. Moreover, evidence from TDP43-mutant mouse models and spinal cord samples from patients with ALS suggests that endogenous levels of TDP43 can release mtDNA into the cytoplasmic fraction via the mitochondrial permeability transition pore, activating the cGAS/STING pathway (Yu et al., 2020). *In vitro* and *in vivo* studies have shown that small-molecule inhibitors targeting STING could prevent TDP43-mediated inflammation and alleviate the symptoms of neuronal decline in TDP43-driven neurodegeneration, indicating that targeting this pathway may be an attractive therapeutic strategy for ALS (Vincent et al., 2017; Haag et al., 2018).

In addition to monogenic inheritance in patients with ALS, several genetic screenings have focused on oligogenic and polygenic inheritance of ALS risk. The subset of sporadic ALS patients with two or more mutation variants is more likely to develop earlier onset, which also showed significant mtDNA alteration in patients with ALS (McCann et al., 2020; Shepheard et al., 2021; He et al., 2024a).

**Mitochondrial DNA disturbances in the pathogenesis of frontotemporal lobe degeneration**

FTLD is the second most common type of young-onset dementia, characterized by progressive cognitive dysfunction, early social-emotional-behavioral changes, difficulties in understanding, primary progressive aphasias, and frequently psychiatric symptoms (Grossman et al., 2023). FTLD shares a partially overlapping spectrum of genetic and neuropathological symptoms with ALS, leading to a mixed disease of FTLD and ALS. Genetic screening has found that FTLD can be caused by multiple pathogenic mutations in C9orf72, FUS, TDP43, microtubule-associated protein tau, and granulin. A body of evidence collectively shows the accumulation of mtDNA single-nucleotide variant (mtSNV) burden in C9orf72 ALS-FTLD human cerebral organoids. The mtDNA single-nucleotide variants within C9orf72-mutant astroglia are more likely to induce mitochondrial dysfunction (**Additional Table 1**). The glial cells can affect mitochondrial homeostasis by altering cellular metabolism and regulating redox stress and Ca^2+^ homeostasis in ALS-FTLD (Nie et al., 2025). Mutations in coiled-coil-helix-coiled-coil-helix domain 10 (CHCHD10) may influence the pathogenesis of FTLD. The CHCHD genes are nuclear-encoded but code for mitochondrial proteins that are highly conserved in their physiological functions. Deletions or mutations in the *CHCH* gene can induce mitochondrial dysfunction. Increasing evidence suggests that CHCHD10 is closely related to cognitive disorders and motor neuron diseases in neurodegenerative disorders. In FTLD-TDP human brains and TDP43-mutant transgenic mice, TDP43 can lead to a reduction in CHCHD10 and disrupt its ability to bind both optic atrophy 1 (OPA1) and mitofilin (Liu et al., 2020). Conversely, FTLD-ALS-associated CHCHD10 mutations can result in the mislocalization of TDP43 in the cytoplasm, leading to abnormal mitochondrial transport and compromised synaptic integrity (Woo et al., 2017).

### Mitochondrial DNA disturbances in the pathogenesis of multiple sclerosis

MS is a chronic inflammatory neurological disorder characterized by demyelination and mitochondrial dysfunction. Following demyelination, there is a compensatory response known as the “axonal mitochondrial response to demyelination,” where axonal mitochondria increase in size, number, and activity, contributing to MS-associated neurodegeneration (Patergnani et al., 2017; Mancini et al., 2019; Chen and Mortha, 2025). Mitochondrial dysregulation may lead to excessive secretion of ROS and reactive nitrogen species, playing a role in the pathogenesis of MS. mtDNA, recognized as a pro-inflammatory damage-associated molecular pattern molecule, has been identified as an early biomarker of MS disease activity and treatment response (Fyfe, 2024). Evidence suggests that mtDNA variations, such as mtDNA T4216C and nt13708 G/A variations, may contribute to susceptibility to the clinical progression of certain subgroups of patients with MS (Yu et al., 2008; Andalib et al., 2016; Bailey et al., 2021; Pienaar et al., 2021). Leurs et al. (2018) found a significant increase in CSF mtDNA concentration in 40 cases of progressive MS. MS patients treated with fingolimod showed nearly a 50% decrease in mtDNA copy number concentrations during follow-up compared to baseline, indicating a potential role for mtDNA as an indicator of treatment response (Leurs et al., 2018). Autologous hematopoietic stem cell transplantation (AHSCT) is a promising therapeutic treatment for MS (Muraro et al., 2017). AHSCT is significantly effective in inhibiting inflammation in the CNS and suppressing clinical and MRI-identified MS disease activity. Pavlovic et al. (2024) evaluated CSF mtDNA concentrations after AHSCT treatment and found that the median concentration decreased, regardless of prior disease-modifying treatment (**Additional Table 1**). Taken together, mtDNA in CSF may serve as a potential biomarker for inflammatory activity and treatment response in MS.

## Targeting Mitochondrial DNA Disturbances in the Treatment of Neurodegenerative Diseases

mtDNA disturbances promote the pathobiology of neurodegenerative diseases, suggesting that developing mtDNA-targeted therapeutics could be a promising treatment for these conditions (Song et al., 2024a). The levels of mtDNA mutations and deletions are higher than those of nuclear DNA mutations, which are considered drivers of aging and neurodegenerative diseases. Therefore, it is essential to develop appropriate gene editing tools to rectify abnormal mtDNA alterations and correct heteroplasmic mtDNA disorders. The classical gene editing tool, CRISPR/Cas, has shown limited evidence of effectiveness in editing mtDNA in mammalian cells. However, several engineered nucleases designed for mitochondrial targeting, including mitochondrial-targeted zinc-finger nucleases (mitoZFNs) and mitochondrial-targeted transcription activator-like effector nucleases (mitoTALENs), have been developed to edit mutated mtDNA (Minczuk et al., 2010; Bacman et al., 2013, 2024; Gammage et al., 2014). Recently, an interbacterial toxin called cytidine deaminase toxin (DddA) has been reported to catalyze cytidine deamination, converting cytosine to uracil at desired sites. The uracil glycosylase inhibitor produces RNA-free DddA-derived cytosine base editors (DdCBEs) that convert C-G base pairs to T-A base pairs in mtDNA (Mok et al., 2020; Qiu et al., 2024; Xie et al., 2024).

**Figure 4:**
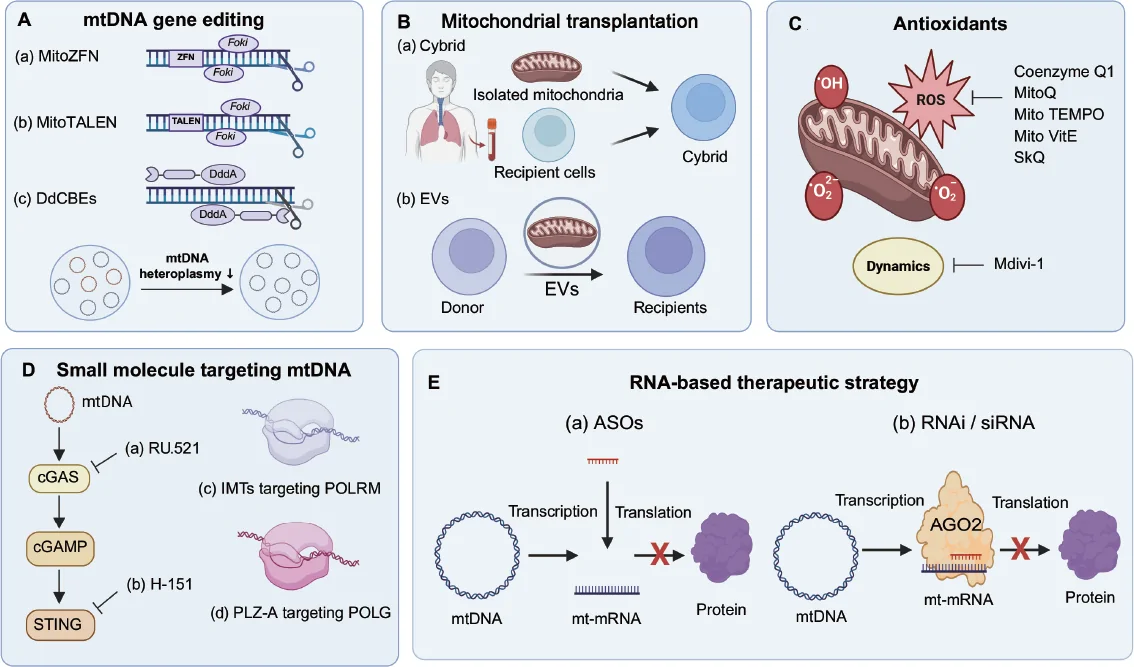
Potential mtDNA-based strategies for treating mtDNA disturbance in neurodegenerative diseases. (A) mtDNA gene editing by mitoZFN (a), mitoTALEN (b), and DdCBEs (c). (B) Mitochondrial transplantation or transfer. (a) cybrid; (b) EVs. (C) Antioxidants of mitochondrial diseases. (D) Small molecule targeting mtDNA. (a) RU.521; (b) H-151; (c) IMTs targeting POLRM; (d) PLZ-A targeting POLG. (E) RNA-based therapeutic strategy by ASOs (a) and RNAi agents (b). Created with BioRender.com. AGO2: Argonaute-2; ASOs: antisense oligonucleotides; cGAMP: cyclic GMP-AMP; cGAS: the cyclic GMP–AMP synthase; DdCBEs: RNA-free DddA-derived cytosine base editors; EVs: extracellular vesicle; mitoTALEN: mitochondrial-targeted transcription activator-like effector nucleases; mitoZFN: mitochondrial-targeted zinc-finger nucleases; POLG: mitochondrial polymerase γ; POLRM: mitochondrial RNA polymerase; STING: stimulator of interferon genes.

In human cells, the editing efficiency of DdCBEs ranges from 4.6% to 49%, with several advantages, including high specific targeting affinity and the ability to target double-stranded DNA without producing DNA strand breaks. Silva-Pinheiro et al. (2022) developed a tool for the *in vivo* delivery of DdCBEs using adeno-associated virus vectors (AAV) (**Figure 4**). The DdCBE system represents a promising strategy for mtDNA editing to rectify abnormal somatic mutations in diseases (**Additional Table 2**).

**Additional Table 2:** Mitochondria-targeted therapeutics for neurodegenerative diseases

Treatment	Mechanism	Potential impact	Model	Reference
mitoZFNs	mtDNA gene editing with *FokI* nuclease	Selective degradation of pathogenic mitochondrial genomes bearing large-scale deletions or point mutations	143B/206 line and fibroblast from patient	Gammage et al., 2014
mitoTALENs	mtDNA gene editing	Restore mtDNA heteroplasmy balance in cells derived from patients	143B/206 line and fibroblast from patients	Bacman et al., 2024
DdCBEs	mtDNA gene editing	Catalyze C•G-to-T•A conversions in human mtDNA with high target specificity and product purity	HEK293T cells, U2OS cells, and primary fibroblasts	Mok et al., 2020
Mitochondrial transplantation	Mitochondrial transfer	Help the cells functional oxygen consumption and respiration in PD	Donor from HepG2 cells	Chang et al., 2016
Pep-1-mediated mitochondrial transplantation	Mitochondrial transfer	Help the cells functional oxygen consumption and respiration in PD	Donor from PC12 cells	Chang et al., 2016
Mitochondrial transplantation	Mitochondrial transfer	Help the cells functional oxygen consumption and respiration in AD	Donor from HeLa cells	Herbert and Turnbull, 2018
Coenzyme Q10 idebenone	Mitochondria-targeted antioxidants	Idebenone nanorod formulations reduced cognitive impairment *in vivo*	Mice induced by Aβ_1-__42_	Boyuklieva et al., 2024
MitoQ	Mitochondria-targeted antioxidants	Decrease ROS level and reduce cognitive decline and AD-like pathology	3xTg-AD mice	Young and Franklin, 2019
MitoTEMPO	Mitochondria-targeted antioxidants	Mitigate Aβ-induced mtDNA depletion	Aβ-induced cell model	Bagheri et al., 2023
MitoVitE	Mitochondria-targeted antioxidants	Reduce caspase activation and oxidative stress	*In vitro* cell model	Bagheri et al., 2023
SkQ	Mitochondria-targeted antioxidants	Reduce ROS production and inhibit the hyperphosphorylation of Aβ_1-42_ and APP	Senescence-accelerated OXYS rats	Bagheri et al., 2023
SS-31	Mitochondria-targeted antioxidants	Reduce ROS production and reduce memory impairment	LPS-induced mice	Ding et al., 2021
Mdivi-1	Mitochondria-targeted antioxidants	Regulate mitochondrial dynamics, ATP production and reduce Aβ deposition	APP/PS1 mice	Bordt et al., 2022
RU.521	cGAS inhibitor	Selective inhibition of cGAS dsDNA-dependent enzymatic activity *in vitro* and in cells	Raw cell lines and Trex1^-/-^ offspring	Vincent et al., 2017
H-151	STING inhibitor	Attenuate autoinflammatory response in cells and in mice	HEK293T cells and mice	Haag et al., 2018
IMTs	POLRM inhibitor	Impair mtDNA transcription and induce a strong anti-tumor response	HeLa cells, A2780 cells, and C57 mice	Bonekamp et al., 2020
PZL-A	POLG proactivator	Promote OXPHOS machinery and cellular respiration	Fibroblasts from patients with POLG	Valenzuela et al., 2025
ASOs or RNAi drug	mtRNA-based rectify	Nano-carriers were constructed to deliver let-7b to mitochondria to reduce mtDNA COX1 and COX2 expression	A542 cell line	Kawamura et al., 2019

AD: Alzheimer's disease; ASO: antisense oligonucleotides; cGAS: cyclic GMP-AMP synthase; DdCBEs: DddA-derived cytosine base editors; mitoTALENs: mitochondrial-targeted transcription activator-like effector nucleases; mitoZFNs: mitochondrial-targeted zinc-finger nucleases; PD: Parkinson's disease; POLG: mitochondrial polymerase γ; POLRM: mitochondrial RNA polymerase; STING: stimulator of interferon genes.

Mitochondrial transfer/transplantation is a novel strategy to improve mtDNA homeostasis (**Additional Table 2**). This strategy originated from the cytoplasmic hybrid cell model (cybrid), which is constructed by fusing mtDNA-depleted (rho0) cells with mtDNA from platelet donors of patients with AD. The early form of mitochondrial replacement therapy was developed through the ooplasmic transplantation of healthy donor mtDNA into recipient oocytes that contained pathogenic mtDNA mutations (Herbert and Turnbull, 2018). In addition to mitochondrial replacement therapy, another promising approach is the administration of purified mitochondria or extracellular vesicle-associated mitochondria as therapeutic cargo to patients (**Figure 4**). This method has been used to treat inherited mitochondrial diseases such as Leigh syndrome. Peptide-mediated allogeneic mitochondrial delivery also shows therapeutic potential by facilitating the incorporation of Pep-1-modified allogeneic mitochondria into the medial forebrain bundle of PD rats. As a result, neuronal viability and locomotor activity are restored through the successful uptake of modified mitochondria in PD rats (Chang et al., 2016).

Oxidative stress and accumulated ROS production are the most common risk factors affecting mitochondrial homeostasis, leading to mtDNA alterations during the aging process. Mitochondria-targeted antioxidants are one type of therapy for neurodegenerative diseases due to their role in mitigating oxidative stress and preventing mtDNA damage (**Additional Table 2**). These antioxidants, including coenzyme Q10, idebenone, MitoQ, MitoTEMPO, MitoVitE, and SkQ, have demonstrated considerable improvements in mitochondrial function and Aβ-associated pathology, thereby preventing cognitive impairment and neurodegeneration in models of AD and PD (McManus et al., 2011; Young and Franklin, 2019; Huang et al., 2021; Rauchova, 2021; Bagheri et al., 2023; Boyuklieva et al., 2024; Pszczolowska et al., 2024). The antioxidant tetrapeptide SS-31, also known as MTP-131, has been shown to improve neuronal dysfunction and memory impairment in lipopolysaccharide-induced mice and in an AD mouse model (Zhao et al., 2019a; Ding et al., 2021). Oxidative stress can impair mitochondrial dynamics by regulating the mitochondrial network, including fusion, fission, and mitophagy (Chen et al., 2010). Mitochondrial division inhibitor-1 (Mdivi-1) is an indirect Drp1 inhibitor that increases the expression of mitochondrial fusion-related genes while decreasing fission genes. Wang et al. (2017) found that Mdivi-1-treated mice exhibited rescued mitochondrial fragmentation and improved mitochondrial function in CRND8 neurons, both *in vitro* and *in vivo*. Additionally, Aβ accumulation and cognitive deficits were prevented following Mdivi-1 treatment (Baek et al., 2017; Bordt et al., 2022; **Figure 4**).

Since abnormal alterations in mtDNA expression occur during aging and in a range of neurodegenerative diseases, extensive efforts have focused on the development of small-molecule therapies targeting mtDNA stability for these conditions (Russell et al., 2020; **Additional Table 2**). Several biotech companies have committed themselves to screening, discovering, and refining mtDNA-associated therapeutics. Recent studies have led to the development of various cGAS and STING inhibitors for the treatment of neurodegenerative diseases. RU.365 and its benzothiazole analog RU.332 were identified as inhibitors of dsDNA-induced cGAS activity. Following a series of chemically synthesized derivatives, RU.521 was designed as a nanomolar cGAS inhibitor (Vincent et al., 2017). Progression of counter and validation screens identified H-151 as a covalent STING antagonist. Pretreatment with H-151 significantly inhibited cytokine responses in both human cells and *in vivo* (Haag et al., 2018). Bonekamp et al. (2020) developed the first-in-class specific inhibitors of mitochondrial transcription that selectively target POLRM and inhibit mtDNA transcription in cells. More recently, their group reported the novel discovery and characterization of PZL-A, a small molecule that can restore mutant POLG activity. PZL-A is capable of binding to the allosteric site of the POLGA-POLGB interface, stimulating POLG processivity. It has been well established that PZL-A is well tolerated and restores mtDNA repopulation rates and OXPHOS activity in fibroblasts isolated from patients with POLG disorder (Valenzuela et al., 2025; **Figure 4**). Although these small molecules are currently applied to improve primary mitochondrial diseases such as Leigh syndrome, encephalomyopathy, and Kearns-Sayre syndrome, their potential application could also be expanded to a broader range of syndromes involving mitochondrial dysfunction.

RNA-based therapeutic strategies, including the delivery of antisense oligonucleotides, RNAi drugs, and mitochondrial microRNA mimics, have been used to rectify mitochondrial DNA (mtDNA) gene expression for the treatment of mitochondrial diseases (**Figure 4**). Kawamura et al. (2019) used a liposome-based nanocarrier known as MITO-porter to deliver therapeutic agents into the mitochondrial matrix, successfully reducing mtDNA COX2 expression. Maghsoudnia et al. (2020) developed nano-carriers, specifically let-7b-PAMAM (G5)-TPP and let-7b-PAMAM (G5)-TPP-HA, to facilitate the delivery of let-7b to mitochondria, which in turn decreased mtDNA COX1 and COX2 expression in the A542 cell line (**Additional Table 2**). Furthermore, functional mRNAs, rRNAs, and tRNAs can be delivered to the mitochondrial matrix to compensate for mtDNA gene expression deficiencies. Mahato et al. (2011) created a polycistronic RNA that included mtDNA-encoded genes and the assembly subunit of the RNA import complex R8 to restore mitochondrial function in Kearns-Sayre syndrome. Although RNA-based therapeutic agents targeting mtDNA show significant therapeutic potential, they have not yet been approved for clinical trials. Numerous obstacles hinder the manipulation of mtDNA with RNA-based therapeutics. The electrochemical gradient and double-membrane architecture of mitochondria pose challenges for transmembrane transport from the outer mitochondrial membrane to the mitochondrial matrix. Moreover, the fundamental mechanisms of RNA import into mitochondria remain poorly understood. It is crucial to gain a deeper understanding of these mechanisms and to evaluate the safety and efficacy of RNA-based therapeutic agents.

Although there is great excitement surrounding more than 50 clinical trials of mitochondria-based therapeutics, which are being considered for the future treatment of primary mitochondrial diseases, several challenges hinder their translation into clinical application. Currently, many of these trials have not provided sufficient scientific data or evidence to gain approval from the US Food and Drug Administration or the European Medicines Agency for mitochondrial diseases (Russell et al., 2020). Given the presence of the blood-brain barrier, both small molecules and gene rectification methods require nanoparticles or intraocular AAV vectors for effective drug delivery and proper guidance into the mitochondrial matrix within the CNS. However, due to low uptake efficiency and off-target effects in cells, many mitochondria-targeting nanocarriers have not yet been approved for clinical use (Yamada et al., 2020). In many cases, there is insufficient evidence of AAV delivery into the CNS. Although the AAV9 serotype can cross the blood–brain barrier, it still faces challenges in effectively targeting neuronal or other glial cells (Zhang et al., 2022a). Additionally, controlling healthy margins and limiting toxicities within an appropriate therapeutic window is difficult due to the high-dose administration often required for AAV applications (Hudry and Vandenberghe, 2019). This situation indicates the need for broader exploration of mitochondrial import determinants.

## Limitations

mtDNA encodes 2 rRNAs, 22 tRNAs, and 13 polypeptides, which are essential for OXPHOS function. Pathological alterations in mtDNA can lead to mtDNA instability, further affecting mitochondrial homeostasis and disrupting cellular energy production. Due to the high energy demand of neurons in the CNS, accumulated mtDNA alterations in neuronal cells during the aging process may contribute to the pathogenesis of neurodegenerative diseases. In this review, we summarize relevant research and reviews on mtDNA alterations in a range of neurodegenerative diseases; however, there are some limitations. First, we primarily focus on six types of neurodegenerative diseases: AD, PD, HD, ALS, FTLD, and MS. Due to limited research, many other types of neurodegenerative diseases have not been fully included. Second, we focus only on data from clinical cases or animal studies, resulting in a lack of connection between clinical data and animal experiments, which is crucial for translational medicine research. Additionally, the relative mtDNA alterations associated with the same pathological background vary between studies. This variability may arise from differences in disease stages, sample sizes, and detection methods. Lastly, there is a paucity of clinical data regarding therapeutics applied to neurodegenerative diseases such as AD, PD, and HD. The potential therapeutics mentioned still face significant obstacles concerning their efficacy and safety. A better understanding of the underlying mechanisms is essential for developing novel therapeutics.

## Conclusion and Future Perspective

In this review, we elaborate on the processing and key proteins related to mtDNA replication, mtDNA transcription, mtRNA translation, mtRNA surveillance, and mtDNA degradation, and particularly emphasize relationships between mtDNA homeostasis and neurodegenerative diseases. Disturbed mitochondrial genomic homeostasis, especially the individual mtDNA variability caused by mtDNA mutations and depletion, plays an essential role in the etiology of neurodegenerative diseases, including AD and PD. The disturbed mtDNA homeostasis mediated by mtDNA mutations and deletion leads to the reduction of ATP production in neuronal cells, which aggravates the pathological processes of neurodegenerative diseases. In recent decades, advances have been made to elucidate the role of mtDNA in the development of neurodegenerative diseases and how mtDNA affects the phenotype onset during aging. To gain a better understanding of the mtDNA disability in the development of aging and neurodegenerative diseases, there are multiple critical gaps of knowledge remaining to be further investigated. Determining the causal relationship between mtDNA disturbances and neurodegenerative diseases is challenging. How do alterations in mtDNA copy number translate from the cellular level to the organ level and even to the behavioral level? Can mtDNA haplogroups be used as biomarkers for clinical prediction? The thresholds for reduced mtDNA copy number vary across different detection methods, tissues, and cell subtypes. Therefore, it is important to define standardized biochemical thresholds and apply single-cell sequencing to create an ultradeep map of the mitochondrial genome. Novel strategies targeting mtDNA offer a precision medicine approach to enhance mitochondrial function in neurodegenerative diseases. However, many of these approaches have not yet been approved for clinical applications. The precise mechanisms underlying the replacement of pathogenic mtDNA and the safety of mitochondrial gene therapy require further investigation and clinical data. Furthermore, treatments targeting mtDNA disturbances should adhere to personalized precision medicine due to individual variability in mtDNA, heteroplasmy, and haplogroups in neurodegenerative diseases. This review suggests that targeting mtDNA homeostasis holds therapeutic potential for neurodegenerative diseases in clinical practice and provides a novel therapeutic target for drug design and screening.

## Additional files:

***Additional Table 1:*** *mtDNA alterations in neurodegenerative disease.*

***Additional Table 2:*** *Mitochondria-targeted therapeutics for neurodegenerative diseases.*

## Data Availability

*All relevant data are within the paper and its Additional files.*
